# AMEERA-4: a randomized, preoperative window-of-opportunity study of amcenestrant versus letrozole in early breast cancer

**DOI:** 10.1186/s13058-023-01740-2

**Published:** 2023-11-10

**Authors:** Mario Campone, François-Clément Bidard, Patrick Neven, Lei Wang, Bin Ling, Yvonne Dong, Gautier Paux, Christina Herold, Ugo De Giorgi

**Affiliations:** 1 Institut de Cancérologie de l’Ouest, René Gauducheau, Boulevard Jacques Monod, 44805 Saint-Herblain, France; 2 Institut Curie, Paris and Saint-Cloud, France; 3 Versailles Saint Quentin, Saint-Cloud, France; 4 Paris-Saclay University, Saint-Cloud, France; 5 Department of Gynaecological Oncology, Multidisciplinary Breast Center, University Hospitals Louvain, Campus Gasthuisberg, Leuven, Belgium; 6 Sanofi, Cambridge, MA USA; 7 Sanofi, Shanghai, China; 8 Sanofi, Beijing, China; 9 Department of Medical Oncology, IRCCS Istituto Romagnolo per lo Studio dei Tumori (IRST) “Dino Amadori”, Meldola, Italy; 10 Present Address: Takeda, Cambridge, MA USA

**Keywords:** Amcenestrant, Early breast cancer, Ki67, Postmenopausal, Window-of-opportunity study

## Abstract

**Background:**

Window-of-opportunity (WOO) studies provide insights into the clinical activity of new drugs in breast cancer.

**Methods:**

AMEERA-4 (NCT04191382) was a WOO study undertaken to compare the pharmacodynamic effects of amcenestrant, a selective estrogen receptor degrader, with those of letrozole in postmenopausal women with newly diagnosed, operable estrogen receptor–positive, human epidermal growth factor receptor 2−negative (ER+/HER2−) breast cancer. Women were randomized (1:1:1) to receive amcenestrant 400 mg, amcenestrant 200 mg, or letrozole 2.5 mg once daily for 14 days before breast surgery. The primary endpoint was change in Ki67 between baseline and Day 15 (i.e., day of surgery).

**Results:**

Enrollment was stopped early because of slow recruitment, in the context of the COVID-19 pandemic. The modified intent-to-treat population consisted of 95 study participants with baseline and post-treatment Ki67 values, whereas the safety population included 104 participants who had received at least one dose of study medication. Relative change from baseline in Ki67 was − 75.9% (95% confidence interval [CI] − 81.9 to − 67.9) for amcenestrant 400 mg, − 68.2% (− 75.7 to − 58.4) for amcenestrant 200 mg, and − 77.7% (− 83.4 to − 70.0) for letrozole (geometric least-squares mean [LSM] estimates). Absolute change in ER H-score from baseline (LSM estimate) was − 176.7 in the amcenestrant 400 mg arm, − 202.9 in the amcenestrant 200 mg arm, and − 32.5 in the letrozole arm. There were no Grade ≥ 3 treatment-related adverse events.

**Conclusions:**

Both amcenestrant and letrozole demonstrated antiproliferative activity in postmenopausal women with previously untreated, operable ER+/HER2− breast cancer and had good overall tolerability.

***Trial Registration*:**

ClinicalTrials.gov, NCT04191382 https://clinicaltrials.gov/ct2/show/NCT04191382. Registered 9 December 2019.

**Supplementary Information:**

The online version contains supplementary material available at 10.1186/s13058-023-01740-2.

## Background

Among women in the United States (US), breast cancer is the most commonly diagnosed malignancy and the second most common cause of cancer mortality [1, 2]. In recent decades, the prognosis of breast cancer has been transformed by earlier detection, due to public health initiatives such as national screening programs, and improvements in disease management. In the US, age-standardized mortality due to breast cancer decreased from 22.6 deaths per 100,000 in 1985 to 12.9 deaths per 100,000 in 2017 [3] and continues to decline.

However, breast cancer is heterogeneous. Outcomes in patients diagnosed with early disease depend on a number of established prognostic and predictive factors such as hormone receptor status, human epidermal growth factor receptor 2 (HER2) status, tumor size, histologic grade, lymph node involvement, age, and molecular subtype [4]. Approximately 80% of breast cancer cases are estrogen receptor–positive (ER+) [5], and most of these are classed as HER2–negative. The prognosis of ER+/HER2− early breast cancer is favorable [6], with 5-year overall survival rates > 90% among US women [7].

Window-of-opportunity (WOO) studies are nontherapeutic drug trials in which patients with newly diagnosed, early-stage breast cancer receive an investigational drug (alone or in combination with other drugs) for several days or weeks during the ‘window’ between diagnosis and primary surgery or neoadjuvant therapy [8, 9]. Such studies are often undertaken to investigate the pharmacodynamic effects of a potential new treatment in patients with previously untreated breast cancer [8, 9].

Changes in one or more biomarkers during the course of the study can be used to assess drug activity. The most commonly used biomarker in WOO studies is Ki67, which is a marker of cellular proliferation and a useful (although imperfect) predictor of treatment benefit and long-term survival outcomes [8]. The results of the POETIC trial established that changes in Ki67 over 14 days predict the effectiveness of novel endocrine therapies for breast cancer [10].

In studies of compounds that modulate ER signaling, it is also common to quantify their effects on ER, and sometimes also on progesterone receptors (PgRs). Although WOO studies do not replace trials that have clinical endpoints, they provide valuable data that may facilitate drug development and assist in clinical decision making [8].

Amcenestrant (SAR439859) is a novel, optimized, oral, selective ER degrader (SERD) that antagonizes and degrades ERs [11]. Here, we present the results of the AMEERA-4 trial, a WOO study undertaken to assess the pharmacodynamic properties of amcenestrant in postmenopausal women with ER+/HER2− early breast cancer.

## Methods

### Study design and conduct

AMEERA-4 (NCT04191382) was an international, prospective, open-label, randomized Phase 2 WOO study conducted at 32 centers in 8 countries (Belgium, France, Italy, Japan, Russia, Spain, Ukraine, and the USA). The study consisted of a screening period, randomization, a 14-day treatment period, and a 30-day safety follow-up period (Additional file 1: Figure S1). The primary objective of the study was to determine whether amcenestrant, given at two different doses, had greater antiproliferative activity than that of letrozole in women with early breast cancer.

The study was conducted in accordance with the Declaration of Helsinki, the Council for International Organizations of Medical Sciences (CIOMS) International Ethical Guidelines, applicable International Conference on Harmonisation Good Clinical Practice Guidelines, and applicable laws and regulations. All study participants provided voluntary written informed consent to participate. No interim analyses were planned; however, the protocol allowed for the study to be terminated at any time, and for any reason, by the sponsor or its designee.

### Patients

Full details of the inclusion and exclusion criteria are given in Additional file 1: Table S1. Briefly, the study enrolled postmenopausal women with newly diagnosed, ER+/HER2−, localized (stage I, stage II, or operable stage III; tumor size ≥ 10 mm by ultrasound) primary breast cancer who were eligible for upfront breast surgery, and who had an Eastern Cooperative Oncology Group performance status (ECOG PS) of 0 or 1. ER positivity was defined as ≥ 1% tumor cell staining by immunohistochemistry (IHC). Patients also had to have a baseline Ki67 level ≥ 15%, as measured by IHC in a diagnostic biopsy per local assessment.

### Treatment

Eligible study participants were randomized 1:1:1 to receive amcenestrant 400 mg (4 × 100 mg capsules), amcenestrant 200 mg (2 × 100 mg capsules), or letrozole 2.5 mg (1 × 2.5 mg tablet), beginning on Day 1 and ending on Day 14 (Additional file 1: Figure S1). All study medication was administered orally, once daily. The doses of amcenestrant studied in AMEERA-4 were selected following a review of preliminary safety, pharmacokinetic, and pharmacodynamic data from previous studies of amcenestrant in participants with advanced or metastatic breast cancer.

Randomization was achieved using a centralized, interactive telephone- and internet-based response system. Patients were issued a diary on Day 1 and asked to record the time of study drug administration each day. Compliance was assessed by reviewing participant diaries on Day 7 and by counting the number of capsules or tablets returned on Day 14.

Dose modification was not mandated in participants experiencing a Grade 1 or 2 adverse event (AE), with the exception of Grade ≥ 2 elevations in alanine aminotransferase (ALT), in the event of which treatment could be stopped and restarted after recovery to Grade ≤ 1 (or baseline levels).

### Endpoints

The primary endpoint was antiproliferative activity, measured as the change in Ki67 between the baseline and post-treatment (i.e., Day 15) tumor biopsies. Ki67 staining and scoring was assessed by IHC using tissue samples from paired pretreatment (baseline) and post-treatment biopsies and was performed at a central laboratory to minimize bias. Ki67 (and receptor H-scores; see following) was assessed using digitally scanned slides prepared from biopsy tissue. Two pathologists, who worked independently from each other and were blinded to treatment allocation, provided baseline and post-treatment scores for each biopsy pair. Then, for each participant, the means of the two baseline values and the two post-treatment values were computed and used in subsequent analyses.

The key secondary endpoints were: (i) the proportion of participants with a relative decrease in Ki67, between baseline and Day 15, of ≥ 50%; (ii) the absolute change from baseline in ER H-score; and (iii) safety and tolerability, assessed as the incidence and severity of treatment-emergent AEs (TEAEs) and treatment-related AEs (TRAEs).

H-score is an IHC-derived measure of receptor expression in a tissue sample. Briefly, the H-score reflects the percentage of stained cells at each level of staining intensity, which in AMEERA-4 was measured on an interval scale from 0 (no staining) to 3 (most intensely stained). The maximum possible H-score was 300 (i.e., if 100% of cells had an intensity score of 3). The change in H-score between the baseline and post-treatment biopsies was considered to reflect the extent of receptor degradation in biopsy tissue.

TEAEs were defined as AEs that developed, worsened, or became serious during the 14-day treatment period or the 30-day safety follow-up period. AEs were coded according to Medical Dictionary for Regulatory Activities (MedDRA) version 24.0, and severity was assessed using National Cancer Institute Common Terminology Criteria for Adverse Events (NCI-CTCAE), version 5.0.

Four AEs of special interest (AESI) were prespecified in the protocol: pregnancy, symptomatic overdose, Grade ≥ 2 ALT elevation, and photosensitivity. Exploratory endpoints included change in PgR H-score; molecular subtype, as assessed using the Prosigna^®^ gene expression assay (PAM50; Veracyte, South San Francisco, CA, USA); and deoxyribonucleic acid (DNA) mutational profile. In addition, genome-wide transcriptome studies were performed using ribonucleic acid (RNA) sequencing technology to measure the relative abundance of RNA transcripts. These data were used to calculate the cell cycle score signature [12].

In participants randomized to amcenestrant, blood samples were drawn for pharmacokinetic analysis immediately before the last dose was taken (on Day 14) and at 3 h post-dose. Mutations of genes related to cancer and/or response to amcenestrant were analyzed at baseline in tumor biopsies and cell-free DNA (cfDNA). For tumor mutational profiling, DNA extracted from formalin-fixed paraffin-embedded tumor tissue slides underwent whole exome sequencing. For cfDNA extracted from plasma samples, the AVENIO ctDNA expanded panel assay (Roche Diagnostics; Indianapolis, IN, USA) was used to identify genomic aberrations in 77 genes.

### Statistical analysis

The primary endpoint (and secondary pharmacodynamic endpoints) were analyzed in the modified intent-to-treat (mITT) population, which included all randomized study participants who took at least one dose of randomized study medication and had both baseline and post-treatment (centrally assessed) Ki67 values. Safety and tolerability were analyzed in the safety population, which included all randomized study participants who took at least one dose of study medication. Pharmacokinetic variables were analyzed in the pharmacokinetic-evaluable population, which included all participants who received at least one dose of amcenestrant and had at least one evaluable post-treatment plasma concentration. As a result of early termination of trial enrollment, no formal statistical inferences were conducted; only descriptive statistics were provided.

Sample size calculations showed that 40 evaluable participants (i.e., with both baseline and post-treatment Ki67 values) per treatment arm would be needed to achieve 85% marginal power, assuming a geometric mean of reduction of 70% for letrozole and 85% for amcenestrant, and a standard deviation of 1 of the log-fold change at the overall one-sided type I error rate of 2.5% controlled with the Hochberg procedure, based on a one-sided *t* test on the log-transformed data. Assuming a 5% non-evaluable participant rate, the total sample size required was determined to be 126 participants (42 per arm).

Changes in Ki67 were analyzed using a geometric least-squares mean (LSM) approach. Geometric LSMs of the proportional change in Ki67 were based on an analysis of covariance (ANCOVA) model for the log-proportional change on treatment, with treatment and log-transformed Ki67 at baseline as fixed effects, and converted by antilog transformation. The geometric LSM of relative reduction (with 95% confidence interval [CI]) was defined as 1 − geometric LSM of the proportional change.

The proportion of participants with a relative decrease from baseline in Ki67 ≥ 50% was reported using descriptive statistics, and the Clopper-Pearson method was used to calculate the 95% CI. For absolute change in ER H-score from baseline, geometric LSMs (with 95% CIs) were based on an ANCOVA model for the change on treatment, with treatment and ER H-score at baseline as fixed effects. Relative change from baseline in ER H-score was reported using descriptive statistics.

Safety data were summarized descriptively.

## Results

### Impact of COVID-19

Enrollment began in February 2020 but was slower than expected because of the COVID-19 pandemic. To minimize delay in the clinical development of amcenestrant, an unplanned administrative interim analysis was performed after 63 study participants had been randomized. This analysis, which included 55 evaluable participants, provided pharmacodynamic and safety data that were deemed sufficient to enable the strategic development decisions for which AMEERA-4 had been designed and did not change the benefit-risk profile of amcenestrant. Consequently, the decision was taken to terminate the trial before it was fully enrolled.

As a result of early termination of the trial, no formal statistical inferences were conducted; only descriptive statistics were provided.

### Participant disposition

**Fig. 1:**
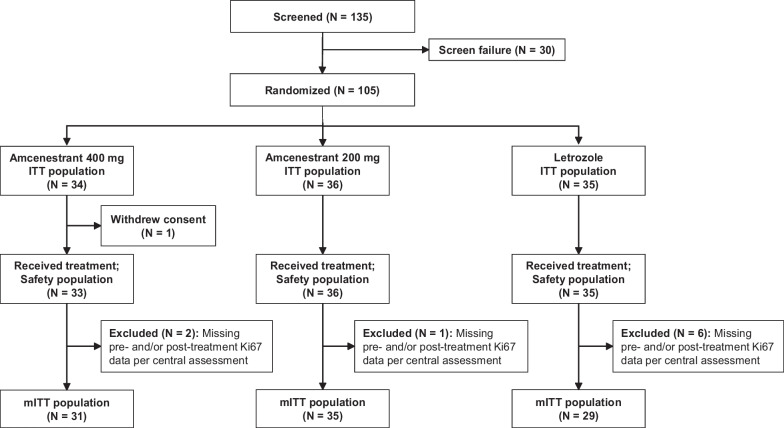
CONSORT diagram. Abbreviations: ITT, intent-to-treat; mITT, modified intent-to-treat

When enrollment was stopped in April 2021, 135 participants had been screened, and 105 had been randomized: 34 to amcenestrant 400 mg, 36 to amcenestrant 200 mg, and 35 to letrozole (Fig. 1). All participants in the amcenestrant 200 mg and letrozole arms of the study completed the treatment period. One participant randomized to receive amcenestrant 400 mg withdrew consent before receiving their first dose of study medication. Thus, there were 104 participants in the safety population. The mITT population included 95 of the 105 randomized participants. The 10 participants who were excluded from the mITT population comprised 9 participants who had incomplete pre- and/or post-treatment Ki67 data (amcenestrant 400 mg: n = 2; amcenestrant 200 mg: n = 1; letrozole: n = 6), and the participant described previously who withdrew consent before receiving any study medication.

### Participant and disease characteristics

The characteristics of the study population were as expected, and there were no major imbalances between the treatment arms (Table 1). In the study population as a whole, median age and body weight were 62.0 years and 71.7 kg, respectively. Most participants were white, and all but three (one in each study arm) had stage I or II breast cancer. In accordance with the inclusion criteria, all participants had luminal B disease (defined as ER+, PgR±, and Ki67 ≥ 15%) by local assessment; 94 (89.5%) had PgR+ tumors.

**Table 1:** Baseline demographics and disease characteristics per local assessment

Parameter	Amcenestrant 400 mg	Amcenestrant 200 mg	Letrozole
No. of study participants	34	36	35
Age (years)	59.5 (49, 85)	63.5 (49, 86)	64.0 (41, 83)
Age categories (years)			
18–64	25 (73.5)	19 (52.8)	20 (57.1)
65–84	8 (23.5)	16 (44.4)	15 (42.9)
≥ 85	1 (2.9)	1 (2.8)	0 (0)
Race			
Asian	2 (5.9)	3 (8.3)	5 (14.3)
Black/African-American	1 (2.9)	0 (0)	0 (0)
White	23 (67.6)	24 (66.7)	25 (71.4)
Multiple	0 (0)	1 (2.8)	1 (2.9)
Missing/not reported	8 (23.5)	8 (22.2)	4 (11.4)
Bodyweight (kg)^a^	72.0 (45, 111)	76.1 (51, 110)	69.4 (48, 118)
ECOG PS^a^			
0	29 (87.9)	31 (86.1)	32 (91.4)
1	4 (12.1)	5 (13.9)	3 (8.6)
Time from diagnosis to randomization (weeks)	3.3 (0, 8)	4.0 (1, 15)	3.9 (1, 8)
Histology			
Ductal adenocarcinoma	25 (73.5)	27 (75.0)	24 (68.6)
Lobular carcinoma	2 (5.9)	3 (8.3)	2 (5.7)
Other carcinoma	6 (17.6)	4 (11.1)	6 (17.1)
Other	1 (2.9)	2 (5.6)	3 (8.6)
Stage			
I	11 (32.4)	13 (36.1)	14 (40.0)
II	22 (64.7)	22 (61.1)	20 (57.1)
IIIA	1 (2.9)	1 (2.8)	1 (2.9)
PgR status			
Positive	28 (82.4)	34 (94.4)	32 (91.4)
Negative	6 (17.6)	2 (5.6)	3 (8.6)
Ki67 (%)	20.0 (15, 80)	25.0 (15, 80)	25.0 (15, 80)
Ki67			
≥ 15% to < 20%	9 (26.5)	6 (16.7)	10 (28.6)
≥ 20%	25 (73.5)	30 (83.3)	25 (71.4)
Tumor size (mm)	22.0 (10, 52)	20.5 (10, 47)	21.0 (10, 63)
Tumor size (mm)^b^			
≥ 10 to < 20	14 (41.2)	15 (41.7)	13 (37.1)
≥ 20	20 (58.8)	21 (58.3)	22 (62.9)

Continuous variables (e.g., age) are presented as median (minimum, maximum) values. Categorical variables are presented as number of study participants (percentage)

^a^Data missing for one study participant in the amcenestrant 400 mg arm

^b^Greatest dimension

*ECOG*, Eastern Cooperative Oncology Group; *PgR*, progesterone receptor; *PS*, performance status

The representativeness of the study population can be evaluated with reference to the information summarized in Additional file 1: Table S2.

Disease characteristics per central assessment are shown in Additional file 1: Table S3. Eleven percent (11/100) of participants across treatment groups had Ki67 < 15% (i.e., were classed as having luminal A disease), with less prevalence in the amcenestrant 200 mg group (2/36 participants; 5.6%) than in the amcenestrant 400 mg group (4/32 participants; 12.5%) or letrozole group (5/32 participants; 15.6%). The proportion of participants with Ki67 ≥ 20% was higher in the amcenestrant 400 mg group (26/32 participants; 81.3%) than in the amcenestrant 200 mg group (26/36 participants; 72.2%) or letrozole group (22/32 participants; 68.8%).

Median ER and PgR H-scores at baseline, per central review, were 300.0 and 120.0, respectively, in the amcenestrant 400 mg arm; 295.0 and 110.0, respectively, in the amcenestrant 200 mg arm; and 299.0 and 172.5, respectively, in the letrozole arm (Additional file 1: Table S3).

### Outcomes

In the safety population (n = 104), the median relative dose intensity (defined as [actual dose intensity/planned dose intensity] × 100, where planned dose intensity [mg/day] = planned dose at Day 1) was 100% in all three treatment arms. Only one episode of dose modification was reported; this was due to participant error and occurred in the 200 mg arm. Three participants (one in the 200 mg arm and two in the 400 mg arm) had a total of four episodes of dose omission (one episode due to COVID-19, two because the participant forgot, and one unexplained). All participants underwent surgery for breast cancer as planned, between one and three days after the last dose of study medication. Ninety-two participants (88.5%) underwent surgery the day after their final dose.

### Pharmacodynamics and pharmacokinetics

**Fig. 2:**
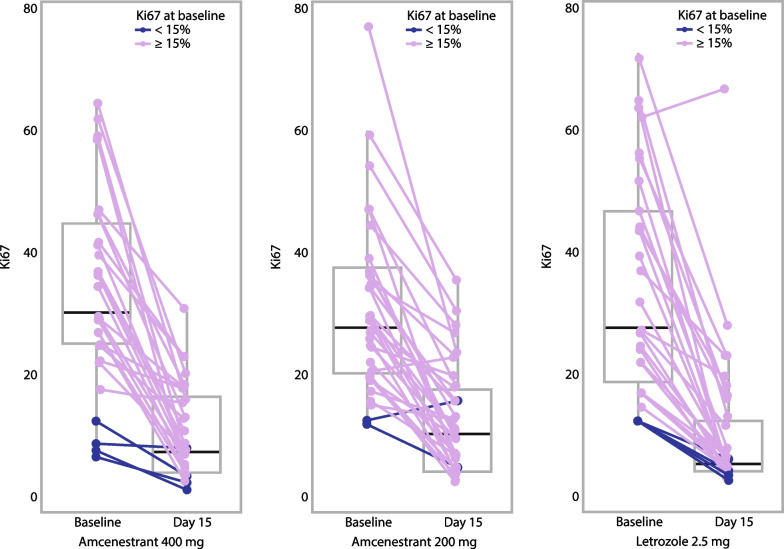
Absolute change in Ki67 from baseline to Day 15 per central review, by baseline Ki67 (mITT population). Each colored line sloping from left to right represents an individual study participant. Reading from top to bottom, the box-and-whisker plots indicate: the highest observation within the range of Q3 and Q3 + 1.5 × (Q3–Q1); Q3; the median value; Q1; and the lowest observation within the range of Q1 and Q1–1.5 × (Q3–Q1). Abbreviations: mITT, modified intent-to-treat; Q, quartile

Decreases in Ki67 expression were observed in most participants in all treatment groups (Fig. 2). Geometric mean Ki67 values at baseline and Day 15, and relative change from baseline, are summarized in Table 2. The geometric LSM estimates for relative change from baseline in Ki67 were − 75.9% (95% CI − 81.9 to − 67.9) for amcenestrant 400 mg, − 68.2% (95% CI − 75.7 to − 58.4) for amcenestrant 200 mg, and − 77.7% (95% CI − 83.4 to − 70.0) for letrozole. The geometric LSM ratio of proportional change versus letrozole was 1.08 (95% CI 0.72–1.63) for amcenestrant 400 mg and 1.42 (95% CI 0.95–2.12) for amcenestrant 200 mg (ratios < 1 favor amcenestrant). The proportion of participants with a ≥ 50% reduction in Ki67 versus baseline was 74.2% (95% CI 55.4–88.1) in the amcenestrant 400 mg arm, 68.6% (95% CI 50.7–83.1) in the amcenestrant 200 mg arm, and 89.7% (95% CI 72.6–97.8) in the letrozole arm. Twelve participants (5 in the amcenestrant 400 mg arm, 3 in the amcenestrant 200 mg arm, and 4 in the letrozole arm) had complete cell cycle arrest (CCCA), defined as post-treatment Ki67 ≤ 2.7%.

**Table 2:** Pharmacodynamic results (mITT population, n = 95)

Parameter	Amcenestrant 400 mg (n = 31)	Amcenestrant 200 mg (n = 35)	Letrozole (n = 29)
Ki67 (%)			
Baseline^a^	29.7	28.6	29.4
Day 15^a^	7.1	9.1	6.6
Relative change (95% CI)^b^	− 75.9 (− 81.9 to − 67.9)	− 68.2 (− 75.7 to − 58.4)	− 77.7 (− 83.4 to − 70.0)
Patients with ≥ 50% reduction in Ki67 from baseline, % (95% CI)	74.2 (55.4 to 88.1)	68.6 (50.7 to 83.1)	89.7 (72.6 to 97.8)
Patients with post-treatment Ki67 ≤ 2.7%, % (95% CI)^c^	16.1 (5.5 to 33.7)	8.6 (1.8 to 23.1)	13.8 (3.9 to 31.7)
ER H-score			
Baseline^d^	286.6 (38.1)	276.8 (37.9)	289.5 (15.1)
Day 15^d^	103.7 (80.2)	84.8 (67.0)	248.8 (54.3)
Absolute change (95% CI)^e^	− 176.7 (− 201.4 to − 152.0)	− 202.9 (− 226.1 to − 179.7)	− 32.5 (− 57.2 to − 7.7)
Relative change (%)^f^	− 65.3 (− 100, 216)	− 68.3 (− 100, 3)	− 9.5 (− 81, 15)
PgR H-score			
Baseline^d^	109.4 (90.1)	121.6 (93.5)	138.1 (102.2)
Day 15^d^	56.6 (70.7)	55.1 (63.4)	38.7 (51.1)
Absolute change (95% CI)^e^	− 58.2 (− 78.4 to − 38.1)	− 68.3 (− 87.4 to − 49.2)	− 88.4 (− 110.1 to − 66.6)
Relative change (%)^f^	− 70.0 (− 100, 5233)	− 74.4 (− 100, 3)	− 75.3 (− 100, 13)

^a^Geometric mean

^b^Geometric least-squares mean (LSM), based on an analysis of covariance (ANCOVA) model for the log proportional change, with treatment and log-Ki67_pre_ as fixed effects and converted by antilog transformation. The geometric LSM of Ki67 reduction is defined as 1 − geometric LSM of the proportional change

^c^Indicating complete cell cycle arrest

^d^Mean (standard deviation)

^e^LSM of absolute change from baseline, based on an ANCOVA model with treatment and baseline as fixed effects. The baseline values of all participants with a change from baseline were used to calculate the LSM

^f^Median (minimum, maximum)

*ANCOVA*, analysis of covariance;*CI*, confidence interval; *ER*, estrogen receptor; *LSM*, least-squares mean; *mITT*, modified intent-to-treat; *PgR*, progesterone receptor

**Fig. 3:**
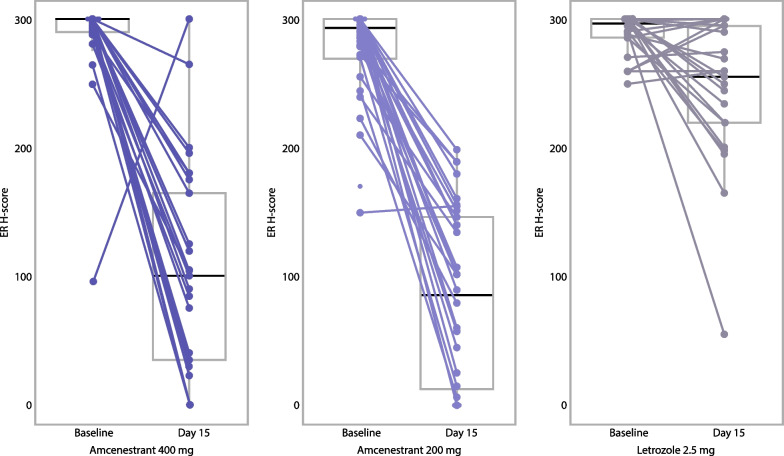
Absolute change in ER H-score from baseline to Day 15 per central review (mITT population). Each colored line sloping from left to right represents an individual study participant. Reading from top to bottom, the box-and-whisker plots indicate: the highest observation within the range of Q3 and Q3 + 1.5 × (Q3–Q1); Q3; the median value; Q1; and the lowest observation within the range of Q1 and Q1–1.5 × (Q3–Q1). Abbreviations: ER, estrogen receptor; mITT, modified intent-to-treat; Q, quartile

Individual changes in ER H-score are shown in Fig. 3. The LSM estimate (95% CI) of the absolute change in ER H-score from baseline, based on central assessment, was − 176.7 (− 201.4 to − 152.0) in the amcenestrant 400 mg arm and − 202.9 (− 226.1 to − 179.7) in the amcenestrant 200 mg arm (Table 2). One participant in the amcenestrant 400 mg arm had a high percentage change in ER H-score (+ 215.8%) that impacted the overall result for the group. The median relative reduction in the ER H-score was 65.3% in the amcenestrant 400 mg arm and 68.3% in the amcenestrant 200 mg arm. In contrast, and as predicted by its mechanism of action, letrozole was associated with modest reductions in ER H-score from baseline to Day 15 (mean absolute change, − 32.5; median relative change, − 9.5%).

PgR H-scores also decreased from baseline to Day 15 in all treatment groups (Table 2 and Additional file 1: Figure S2). The LSM (95% CI) of absolute change from baseline was − 58.2 (− 78.4 to − 38.1) in the amcenestrant 400 mg arm, − 68.3 (− 87.4 to − 49.2) in the amcenestrant 200 mg arm, and − 88.4 (− 110.1 to − 66.6) in the letrozole arm. The median relative changes from baseline were − 70.0%, − 74.4% and − 75.3%, respectively.

Amcenestrant plasma concentrations were measured in 30 participants in the amcenestrant 400 mg arm and 27 participants in the amcenestrant 200 mg arm. On Day 14, geometric mean plasma concentrations were 258 ng/mL at pre-dose, rising to 2228 ng/mL at 3 h post-dose in the 200 mg group, and 452 ng/mL at predose, rising to 3399 ng/mL in the 400 mg group. These concentrations showed an increase of systemic exposure between the 200 mg and 400 mg doses (Additional file 1: Table S4).

### Safety

Sixteen participants (48.5%) in the amcenestrant 400 mg arm, 16 participants (44.4%) in the amcenestrant 200 mg arm, and 18 participants (51.4%) in the letrozole arm experienced at least one TEAE of any grade during the treatment and post-treatment periods (Table 3). Almost all TEAEs were of Grade 1 or 2 severity. Two participants, both of whom were in the amcenestrant 200 mg arm, experienced a TEAE of Grade 3 or higher (one case of pneumonia and one of wound infection). These AEs required hospitalization and were therefore considered serious but were not considered to be treatment related. No participants discontinued treatment due to a TEAE, and there were no deaths either during the treatment or follow-up periods.

**Table 3:** TEAEs and TRAEs reported in ≥ 5% of study participants in one or more study arms

Preferred term, n (%)	Amcenestrant 400 mg (n = 33)		Amcenestrant 200 mg (n = 36)		Letrozole (n = 35)	
	All	Grade ≥ 3	All	Grade ≥ 3	All	Grade ≥ 3
TEAEs						
Any TEAE	16 (48.5)	0	16 (44.4)	2 (5.6)^a^	18 (51.4)	0
Hot flush	4 (12.1)	0	1 (2.8)	0	5 (14.3)	0
Insomnia	4 (12.1)	0	1 (2.8)	0	0	0
Headache	3 (9.1)	0	0	0	2 (5.7)	0
Arthralgia	2 (6.1)	0	0	0	3 (8.6)	0
Asthenia	2 (6.1)	0	2 (5.6)	0	0	0
Decreased appetite	2 (6.1)	0	1 (2.8)	0	0	0
Fatigue	2 (6.1)	0	1 (2.8)	0	1 (2.9)	0
Feeling cold	2 (6.1)	0	0	0	0	0
Procedural pain	2 (6.1)	0	1 (2.8)	0	2 (5.7)	0
ALT increased	1 (3.0)	0	2 (5.6)	0	0	0
Anxiety	1 (3.0)	0	2 (5.6)	0	0	0
Breast pain	1 (3.0)	0	0	0	3 (8.6)	0
Constipation	1 (3.0)	0	2 (5.6)	0	1 (2.9)	0
Diarrhea	0	0	3 (8.3)	0	3 (8.6)	0
TRAEs						
Any TRAE	7 (21.2)	0	8 (22.2)	0	9 (25.7)	0
Hot flush	2 (6.1)	0	1 (2.8)	0	5 (14.3)	0
Headache	2 (6.1)	0	0 (0)	0	1 (2.9)	0
Feeling cold	2 (6.1)	0	0 (0)	0	0 (0)	0
Arthralgia	1 (3.0)	0	0 (0)	0	3 (8.6)	0
Asthenia	1 (3.0)	0	2 (5.6)	0	0 (0)	0
Diarrhea	0 (0)	0	1 (2.8)	0	2 (5.7)	0

^a^Pneumonia (n = 1) and wound infection (n = 1)

*ALT*, alanine aminotransferase;* TEAE*, treatment-emergent adverse event; *TRAE*, treatment-related adverse event

Seven participants (21.2%) in the amcenestrant 400 mg arm, 8 participants (22.2%) in the amcenestrant 200 mg arm, and 9 participants (25.7%) in the letrozole arm experienced at least one TRAE of any grade during the treatment and follow-up periods (Table 3). There were no serious TRAEs in any arm, nor any TRAEs of Grade ≥ 3 severity. The most commonly reported TRAEs (i.e., reported in ≥ 5% of participants in at least one study arm) were hot flush, headache, feeling cold, arthralgia, asthenia, and diarrhea. Hot flush, arthralgia, and diarrhea were reported by more participants in the letrozole arm than in either of the amcenestrant arms. TRAEs reported in a single participant in ≥ 1 treatment arm were abdominal distension, alopecia, constipation, decreased appetite, dry skin, dyspepsia, fatigue, increased ALT, insomnia, lower abdominal pain, musculoskeletal pain, musculoskeletal stiffness, myalgia, nausea, night sweats, pain in extremity, pollakiuria, and upper abdominal pain.

Three participants each had a single pre-specified AESI, all of which were non-serious Grade 2 increases in ALT. All three participants had received amcenestrant: one had received 400 mg, and the other two had received 200 mg. However, only the case at 400 mg was considered to be related to study drug. All cases resolved without sequelae and did not necessitate treatment interruption or dose modification. No bradycardia (pulse rate < 50 beats/min) or eye disorders, other than a single case of dry eye that was unrelated to study treatment, were reported in any study group.

### Genomics and transcriptomics

Cell cycle score signatures decreased in all three treatment groups (Additional file 1: Table S5 and Figure S3). The median change from baseline was − 0.83 in the amcenestrant 400 mg arm, − 0.56 in the amcenestrant 200 mg arm, and − 0.84 in the letrozole arm. Changes in molecular subtype from baseline to Day 15, assessed using the Prosigna^®^ gene expression assay (PAM50), are summarized in Additional file 1: Figure S4. In all three groups, randomized treatment was associated with an increase in the number of participants with luminal A disease and a simultaneous decrease in the number with luminal B disease.

Mutational profiling of tumor DNA was performed at baseline in 75 participants (26 participants in the amcenestrant 400 mg arm, 28 participants in the amcenestrant 200 mg arm, and 21 participants in the letrozole arm [Additional file 1: Figure S5]). The most commonly mutated genes at baseline were *PIK3CA* (36.0%), *TP53* (22.7%), and *GATA3* (17.3%). Mutant *BRCA1* and *BRCA2* were each identified in 4 participants (5%), including one participant who had mutations in both genes. Data on baseline mutations in cfDNA were available for 92 participants, 26 of whom had wild-type DNA and 66 of whom had mutations (24 participants in the amcenestrant 400 mg arm, 23 participants in the amcenestrant 200 mg arm, and 19 participants in the letrozole arm [Additional file 1: Figure S6]). The most common mutations were in the *TP53* and *GNAS* genes (22.7% and 9.1% of participants, respectively).

## Discussion

The findings of AMEERA-4 indicate that both amcenestrant and letrozole have antiproliferative activity in participants with previously untreated ER+/HER2− breast tumors and high baseline Ki67 scores. Most participants had at least a 50% reduction in Ki67 after 14 days’ treatment. The reduction in Ki67 was numerically greater with letrozole compared with that in either of the amcenestrant treatment groups. As predicted by its mechanism of action, amcenestrant produced marked reductions in ER H-scores, whereas letrozole was associated with only minor reductions. PgR H-scores were also reduced, which was expected from the relationship between ER signaling and PgR expression, and consistent with previous findings [13]. Amcenestrant was well tolerated, with only 24 participants experiencing a TRAE of any grade. The most common TRAEs in the amcenestrant arms were hot flush, headache, feeling cold, and asthenia, which were experienced by < 10% of participants and always mild or moderate in intensity. Overall, the results show that amcenestrant had favorable effects on tumor biology at clinically viable doses.

There are several oral SERDs currently in clinical development for ER+/HER2− breast cancer, of which elacestrant (RAD1901), camizestrant (AZD9833), giredestrant (GDC-9545), and imlunestrant (LY3484356) are now in Phase 3 trials. Interim results from the first 46 patients in a WOO study of giredestrant (10 mg, 30 mg [the dose being studied in Phase 3 clinical trials], or 100 mg once daily for 14 days) administered to postmenopausal women with newly diagnosed ER+/HER2− breast cancer showed that, overall, giredestrant reduced tumor Ki67 expression by 79% [14], with no relationship between dose and effect size. ER activity was reduced in 98% of patients who had paired biopsy data (n = 42), with a mean proportional decrease of 79%. Mean proportional reductions in ER and PgR H-scores were 71% and 60%, respectively. In addition, the randomized coopERA breast cancer trial (NCT04436744) included a 2-week WOO phase that compared giredestrant 30 mg with anastrozole 1 mg in postmenopausal women with ER + /HER2− breast cancer [15]. In the primary analysis including 221 patients, the geometric mean relative reduction in Ki67 was greater with giredestrant versus anastrozole (− 75% vs. − 67%; *p* = 0.0433); findings were consistent regardless of baseline Ki67 (≥ 20% or < 20%). CCCA was observed in 20% of tumors treated with giredestrant versus 13% with anastrozole.

Differences in study design and population may explain why giredestrant had a greater antiproliferative effect than anastrozole in coopERA, whereas the effects of amcenestrant and letrozole appeared comparable in AMEERA-4. The choice of comparator (anastrozole in coopERA; letrozole in AMEERA-4) may be relevant, but a previous WOO study in patients with ER+ invasive breast cancer found that anastrozole 1 mg and letrozole 2.5 mg (both once daily) were similarly effective in suppressing Ki67 after 14 days [16]. Of note, Ki67 levels were not part of the eligibility criteria, and baseline Ki67 was low in both groups (anastrozole: 5.8%; letrozole: 6.4%). Additionally,, anastrozole and letrozole appear to have comparable clinical efficacy in the neoadjuvant and adjuvant settings [10, 17, 18] and have been found to reduce Ki67 to a similar extent over 16–18 weeks in the Z1031 neoadjuvant study [18]. Thus, the difference in findings between coopERA and AMEERA-4 may not be explained solely by the choice of aromatase inhibitor.

An alternative explanation is that baseline median Ki67 scores were higher in coopERA than in AMEERA-4 despite a lower inclusion threshold (≥ 5% in coopERA, vs. ≥ 15% in AMEERA-4). Baseline median Ki67 in coopERA was 32.6% among giredestrant recipients and 39.6% among anastrozole recipients [15], while median Ki67 in AMEERA-4 was 25.0% or lower in each of the three study arms. The studies also had different entry requirements with respect to tumor stage and size. Whereas coopERA included patients with either operable or inoperable (stage IIIB or IIIC) breast cancer [19], only those amenable to surgery were included in AMEERA-4. The median baseline tumor size in AMEERA-4 was 21.0 mm (maximum 63 mm). Although the data have not yet been reported, the median tumor size is likely to be larger in coopERA because the inclusion threshold was ≥ 1.5 cm, and patients with T4 tumors could be enrolled [15]. Thus, key differences exist between AMEERA-4 and coopERA, and caution is needed when comparing results from these studies.

WOO studies of camizestrant and elacestrant are ongoing, with no results released to date. SERENA-3 (NCT04588298) is investigating the effects of 5–7 days’ treatment with 3 different doses of camizestrant on ER H-scores and Ki67 in 132 women with ER+/HER2− primary breast cancer [20, 21]. In the ELIPSE trial of elacestrant (400 mg once daily), the primary endpoint is CCCA, and secondary endpoints include tumor subtype and Ki67 [22].

Although WOO studies do not have therapeutic intent, the potential clinical utility of Ki67 response to neoadjuvant endocrine therapy (fulvestrant, anastrozole, or both in combination) in patients with ER+ breast cancer is being investigated in the ALTERNATE (Alliance A011106; NCT01953588) trial [23–25]. In this study, endocrine-resistant tumors are identified early on via Ki67 measurement after 4 and (optionally) 12 weeks of treatment; patients with Ki67 > 10% are switched to neoadjuvant chemotherapy, and efficacy is measured as pathological complete response to treatment [24]. Evidence to date indicates that patients with Ki67 > 10% are unlikely to achieve a pathologic complete response following a switch to neoadjuvant chemotherapy, with more effective treatment strategies needed for these patients [23]. The ALTERNATE trial is also investigating the relationship between recurrence-free survival (RFS) and modified Preoperative Endocrine Prognostic Index (mPEPI) score at surgery (0 vs. > 0), of which CCCA (Ki67 ≤ 2.7%) is a component. Although RFS data are not yet available, fulvestrant (alone or in combination with anastrozole) was not found to be better than anastrozole in terms of the proportion of patients with an mPEPI score of zero at the time of surgery [25].

Our study has several limitations, not least of which is the early termination of the trial and the resulting absence of formal inferential statistical analyses. WOO studies are primarily intended to provide information about the pharmacologic effects of new treatments, and as such no definitive conclusions can be drawn about the clinical efficacy or safety of amcenestrant. Additionally, we studied amcenestrant in postmenopausal women with luminal B-type cancer; thus, the results should not be generalized to premenopausal women or those with other disease subtypes. Lastly, the clinical development of amcenestrant has been discontinued, limiting the applicability of the results. However, the study design also has strengths. Although it was an open-label trial, potential bias was reduced by using centralized randomization and masking the histopathologists who assessed the primary endpoint (Ki67) to treatment allocation. The prospective, controlled trial design provides reassurance that the effects on tumor biology were real rather than chance occurrences. Moreover, the multinational, multicenter design of the study reduced potential bias associated with center-specific factors.

## Conclusions

In conclusion, amcenestrant demonstrated pharmacodynamic activity in women with ER+/HER2− breast cancer, with marked reductions in Ki67 and hormone receptor H-scores. Changes in ER H-score confirm that amcenestrant demonstrates potent ER target engagement and degradation. Additionally, amcenestrant had a favorable safety profile at both dose levels.

However, we did not observe any advantage of amcenestrant over letrozole, a finding consistent with interim data from the Phase 3 AMEERA-5 trial (amcenestrant versus letrozole, both in combination with palbociclib, as first-line treatment for advanced or metastatic ER+/HER2− breast cancer) [26]. Because of these and other findings, the development of amcenestrant as a treatment for breast cancer has been discontinued.

### Supplementary Information

**Additional file 1:** Campone_AMEERA-4 manuscript_Appendix.docx (supplementary tables and figures)

## Data Availability

Qualified researchers may request access to patient-level data and related study documents including the clinical study report, study protocol with any amendments, blank case report form, statistical analysis plan, and dataset specifications. Patient-level data will be anonymized, and study documents will be redacted to protect the privacy of our trial participants. Further details on Sanofi’s data sharing criteria, eligible studies, and process for requesting access can be found at https://www.vivli.org/.

## Abbreviations

ECOG PS: Eastern Cooperative Oncology Group performance status
AE: Adverse event
AESI: Adverse event of special interest
ALT: Alanine aminotransferase
ANCOVA: Analysis of covariance
ASCO: American Society of Clinical Oncology
CCCA: Complete cell cycle arrest
cfDNA: Cell-free DNA
CI: Confidence interval
CIOMS: Council for International Organizations of Medical Sciences
DNA: Deoxyribonucleic acid
ER: Estrogen receptor
HER2: Human epidermal growth factor receptor 2
IHC: Immunohistochemistry
LSM: Least-squares mean
MedDRA: Medical Dictionary for Regulatory Activities
mITT: Modified intent-to-treat
mPEPI: Preoperative Endocrine Prognostic Index
NCI-CTCAE: National Cancer Institute Common Terminology Criteria for Adverse Events
PgR: Progesterone receptor
RFS: Recurrence-free survival
RNA: Ribonucleic acid
SERD: Selective estrogen receptor degrader
TEAE: Treatment-emergent adverse event
TRAE: Treatment-related adverse event
US: United States
WOO: Window-of-opportunity
