# Supplementary material for: AMEERA-4: a randomized, preoperative window-of-opportunity study of amcenestrant versus letrozole in early breast cancer
Source: Breast Cancer Res. 2023 Nov 10;25:141. doi: 10.1186/s13058-023-01740-2 (PMC10638815; doi:10.1186/s13058-023-01740-2)

# Additional file 1: Supplementary materials/appendix

**Table S1.** Inclusion and exclusion criteria in AMEERA-4

| **Inclusion criteria** | **Exclusion criteria** |
| --- | --- |
| 1. Female 2. Age ≥18 years 3. Histological or cytological proven diagnosis of invasive breast adenocarcinoma 4. Localized breast cancer eligible for upfront breast conservative surgery or upfront mastectomy: stage I, stage II, or operable Stage III (excludes T4) 5. Postmenopausal, including women with bilateral oophorectomy or bilateral ovarian ablation via radiotherapy 6. Breast tumor size of ≥10 mm in greatest dimension, measured by ultrasound 7. Estrogen receptor positive, defined as ≥1% tumor cell staining by immunohistochemistry 8. Human epidermal growth factor receptor 2 non-overexpressing, by immunohistochemistry 9. Ki67 level of ≥15% at diagnosis from immunohistochemistry of the tumor based on local laboratory results 10. Eastern Cooperative Oncology Group performance status 0 or 1 11. Capable of giving signed informed consent, including compliance with the requirements and restrictions listed in the informed consent form and protocol | 1. Medical history or ongoing gastrointestinal disorders potentially affecting the absorption of amcenestrant or letrozole 2. Inability to swallow normally and to take capsules or tablets 3. Predictable poor compliance with oral treatment 4. Known active hepatitis A, B, or C, or hepatic cirrhosis 5. Any other cancer 6. Evidence of metastatic spread 7. Treatment with any of the following within 2 weeks (or 5 elimination half-lives, whichever is longest) of the first study treatment administration:    1. Strong cytochrome P450 3A inducers    2. Sensitive substrates of P-glycoprotein    3. Sensitive substrates of breast cancer resistance protein    4. Drugs with the potential to inhibit uridine 5'-diphospho-glucuronosyl transferase 8. Use of hormone replacement therapy within 30 days of randomization 9. Use of any investigational agent within 4 weeks before randomization 10. Prior anticancer treatment, unless completed ≥1 year before inclusion in AMEERA-4 11. Prior local or systemic treatment for the new primary breast cancer currently under investigation 12. Inadequate hematologic function, including neutrophils <1.5 × 10^9^/L; platelets <100 × 10^9^/L; hemoglobin <8.0 g/dL 13. Prothrombin time/international normalized ratio (INR) >1.5 times the upper limit of normal (ULN), or outside the therapeutic range if receiving anticoagulant therapy that would affect the prothrombin time/INR 14. Inadequate renal function with serum creatinine ≥1.5 × ULN, or between 1.0 and 1.5 × ULN with estimated glomerular filtration rate <60 mL/min/1.73m^2^ as estimated using the abbreviated Modification of Diet in Renal Disease formula 15. Liver function parameters with one of the following:     1. aspartate aminotransferase >1.5 × ULN     2. alanine aminotransferase >1.5 × ULN     3. total bilirubin >1.5 × ULN |

Abbreviations: INR, international normalized ratio; ULN, upper limit of normal.

**Table S2.** Representativeness of study participants.

| Cancer type(s)/subtype(s)/ stage(s)/condition | ER+/HER2– breast cancer, stages I-IIIA (operable), postmenopausal |
| --- | --- |
| Sex | All the participants in our study were female. ER+/HER2– breast cancer, like other subtypes of the disease, is a predominantly female disease and is rare in men. Male breast cancer represents only 0.5–1.0% of all cases diagnosed annually. Evidence suggests there are molecular differences between male and female breast cancer. Thus, although our study population is broadly representative of patients with ER+/HER2– disease with regard to sex, our findings cannot be generalized to male breast cancer. |
| Age | It is difficult to find recent population-level data on the mean age, at diagnosis, of women with early-phase ER+/HER2– breast cancer. The median age at diagnosis of breast cancer (all subtypes and stages) is 62 years; in our study, the mean age was 59.5 years in the amcenestrant 400 mg group, 63.5 years in the amcenestrant 200 mg group, and 64.0 years in the letrozole group. In the USA, over 85% of patients with breast cancer are diagnosed with early-stage disease; we therefore think that, with respect to age, our study population is likely to be representative of the general population of postmenopausal women with ER+/HER2– breast cancer. |
| Race/ethnicity | Although HR+/HER2– breast cancer is by far the most common subtype of breast cancer, regardless of ethnicity, most participants in our study were white (72/105; 69%). Physicians should therefore exercise caution when applying the results to non-white populations. In particular, our study included only one black/African-American woman, and therefore our findings cannot be generalized to this population. |
| Geography | The 105 patients included in our study were from eight countries (Belgium [n=8], France [21], Italy [14], Japan [9], Russia [15], Spain [14], Ukraine [11], and the USA [13]). |
| Other considerations | None. |

ER, estrogen receptor; HER2, human epidermal growth factor receptor 2; HR, hormone receptor

**Table S3.** Baseline disease characteristics per central assessment.

| **Parameter** | **Amcenestrant 400 mg** | **Amcenestrant 200 mg** | **Letrozole** |
| --- | --- | --- | --- |
| Ki67 (%) |  |  |  |
| No. of study participants | 32 | 36 | 32 |
| Median (min, max) | 29.8 (6, 65) | 27.4 (12, 77) | 27.4 (9, 72) |
| <15% | 4 (12.5) | 2 (5.6) | 5 (15.6) |
| ≥15% to <20% | 2 (6.3) | 8 (22.2) | 5 (15.6) |
| ≥20% | 26 (81.3) | 26 (72.2) | 22 (68.8) |
| ER H-score^a^ |  |  |  |
| No. of study participants | 32 | 35 | 31 |
| Median (min, max) | 300.0 (95, 300) | 295.0 (150, 300) | 299.0 (250, 300) |
| 0 (H-score 0) | 0 (0) | 0 (0) | 0 (0) |
| Low (1–100) | 1 (3.1) | 0 (0) | 0 (0) |
| Moderate (101–200) | 0 (0) | 2 (5.7) | 0 (0) |
| High (201–300) | 31 (96.9) | 33 (94.3) | 31 (100.0) |
| PgR status |  |  |  |
| No. of study participants | 31 | 36 | 30 |
| Positive | 26 (83.9) | 31 (86.1) | 26 (86.7) |
| Negative | 5 (16.1) | 4 (11.1) | 4 (13.3) |
| Indeterminate | 0 (0) | 1 (2.8) | 0 (0) |
| PgR H-score |  |  |  |
| No. of study participants | 31 | 35 | 30 |
| Median (min, max) | 120.0 (0, 300) | 110.0 (0, 300) | 172.5 (0, 300) |
| 0 (H-score 0) | 5 (16.1) | 4 (11.4) | 4 (13.3) |
| Low (1–100) | 9 (29.0) | 11 (31.4) | 8 (26.7) |
| Moderate (101–200) | 13 (41.9) | 14 (40.0) | 7 (23.3) |
| High (201–300) | 4 (12.9) | 6 (17.1) | 11 (36.7) |
| Molecular subtype by IHC |  |  |  |
| No. of study participants | 31 | 35 | 31 |
| Luminal A | 4 (12.9) | 2 (5.7) | 5 (16.1) |
| Luminal B | 27 (87.1) | 33 (94.3) | 26 (83.9) |

Continuous variables (e.g., H-scores) are presented as median (minimum, maximum) values. Categorical variables are presented as number of study participants (percentage).

^a^All study participants were ER–positive by central review, except for one study participant in the amcenestrant 200 mg arm whose status was indeterminate.

Abbreviations: ER, estrogen receptor; IHC, immunohistochemistry; PgR, progesterone receptor.

**Table S4.** Amcenestrant plasma concentrations on Day 14 by time-point.

| **Plasma concentration (ng/mL)** | **Amcenestrant 400 mg (n = 33)** | **Amcenestrant 200 mg (n = 36)** |
| --- | --- | --- |
| Pre-dose |  |  |
| Number | 30 | 27 |
| Mean (SD) | 607 (400) | 376 (469) |
| Geometric mean (CV %) | 452 (66) | 258 (125) |
| 3 hours post-dose |  |  |
| Number | 30 | 27 |
| Mean (SD) | 4305 (2471) | 2596 (1348) |
| Geometric mean (CV %) | 3399 (57) | 2228 (52) |

Only plasma concentrations collected within the predefined time windows (i.e., within 2 hours before administration for pre-dose samples, and 2–4 hours after administration for 3-hour post-dose samples, are included.

Abbreviations: CV, coefficient of variation; SD, standard deviation.

**Table S5**. Cell cycle score signature, derived from RNA sequencing, in the modified intent-to-treat population.

| **Time-point/Parameter** | | **Amcenestrant 400 mg** | **Amcenestrant 200 mg** | **Letrozole** |
| --- | --- | --- | --- | --- |
| Baseline | No. of study participants | 26 | 32 | 24 |
|  | Median (min, max) | 0.58 (–0.4, 0.8) | 0.49 (–0.7, 0.9) | 0.34 (–0.6, 0.8) |
| Day 15 | No. of study participants | 31 | 32 | 28 |
|  | Median (min, max) | –0.43 (–0.7, 0.6) | –0.29 (–0.8, 0.6) | –0.60 (–0.8, 0.8) |
| Change (baseline to D15) | No. of study participants | 26 | 29 | 23 |
|  | Median (min, max) | –0.83 (–1.2, –0.1) | –0.56 (–1.2, 0.1) | –0.84 (–1.2, –0.1) |

**Figure S1. Design of the AMEERA-4 study, with patient numbers included in the mITT and safety populations.** Abbreviations: ER, estrogen receptor; D, day; HER2, human epidermal growth factor receptor 2; IMP, investigational medicinal product; mITT, modified intent-to-treat; QD, once daily.

**
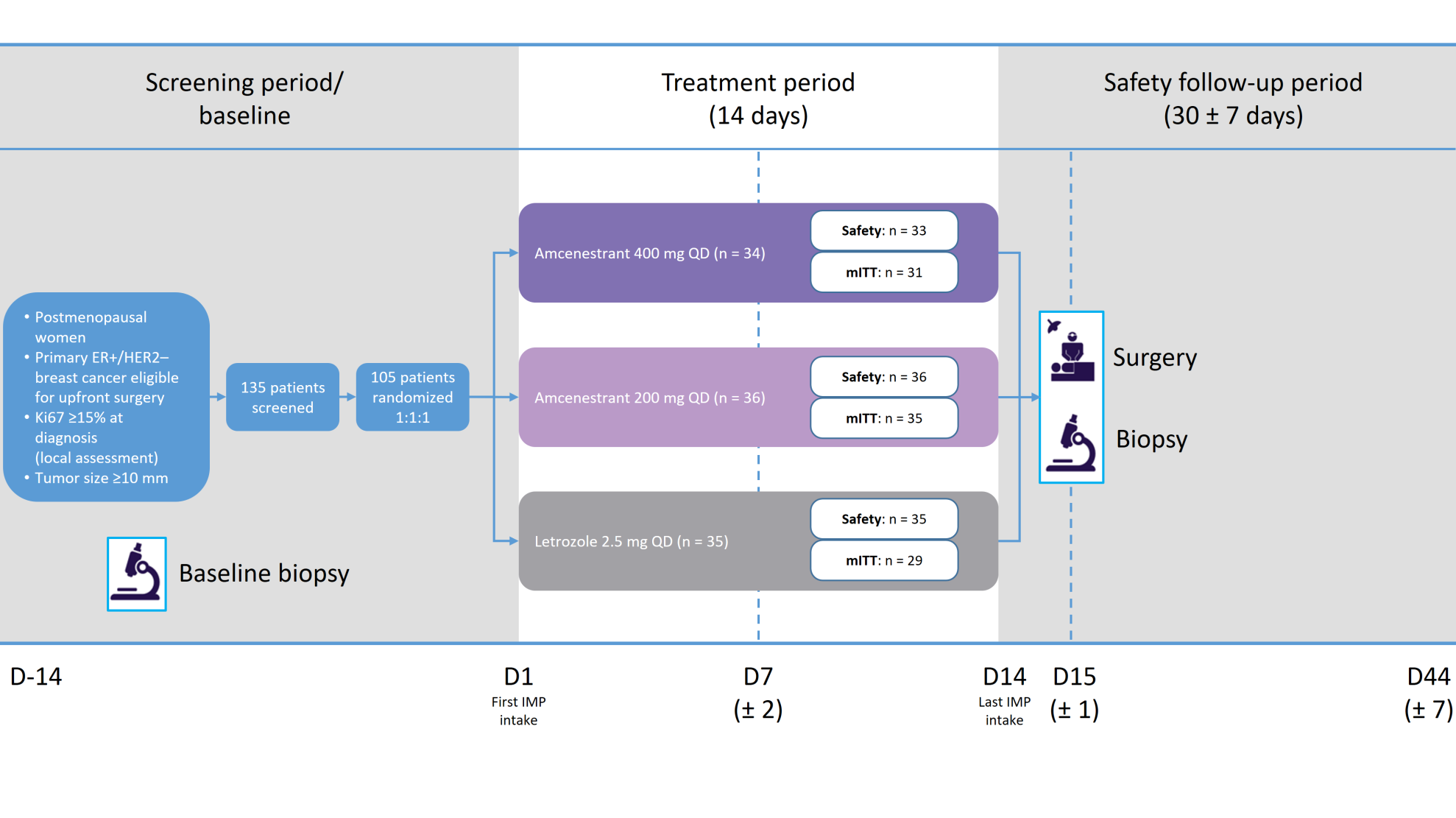
**

**Figure S2.** **Absolute change in PgR H-score from baseline to Day 15 per central review (mITT population).** Each line sloping from left to right represents an individual study participant. Reading from top to bottom, the box-and-whisker plots indicate: the highest observation within the range of Q3 and Q3+1.5×(Q3–Q1); Q3; the median value; Q1; and the lowest observation within the range of Q1 and Q1–1.5×(Q3–Q1). Abbreviation: mITT, modified intent-to-treat; PgR, progesterone receptor; Q, quartile.


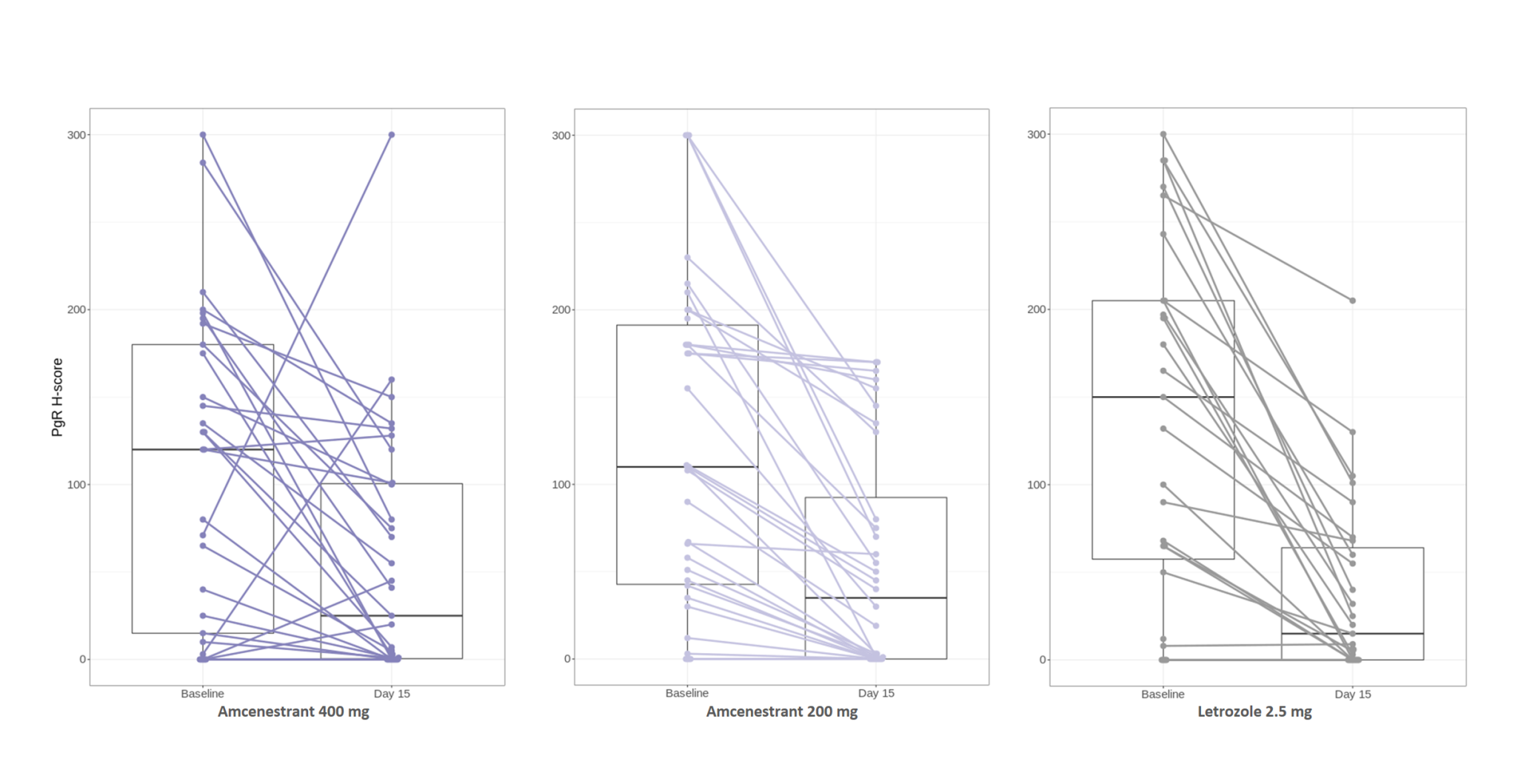


**Figure S3.** **Absolute change in cell cycle score signature from baseline to Day 15 per central review (mITT population).** Each line sloping from left to right represents an individual study participant. Reading from top to bottom, the box-and-whisker plots indicate: the highest observation within the range of Q3 and Q3+1.5×(Q3–Q1); Q3; the median value; Q1; and the lowest observation within the range of Q1 and Q1–1.5×(Q3–Q1). Abbreviation: Q, quartile.


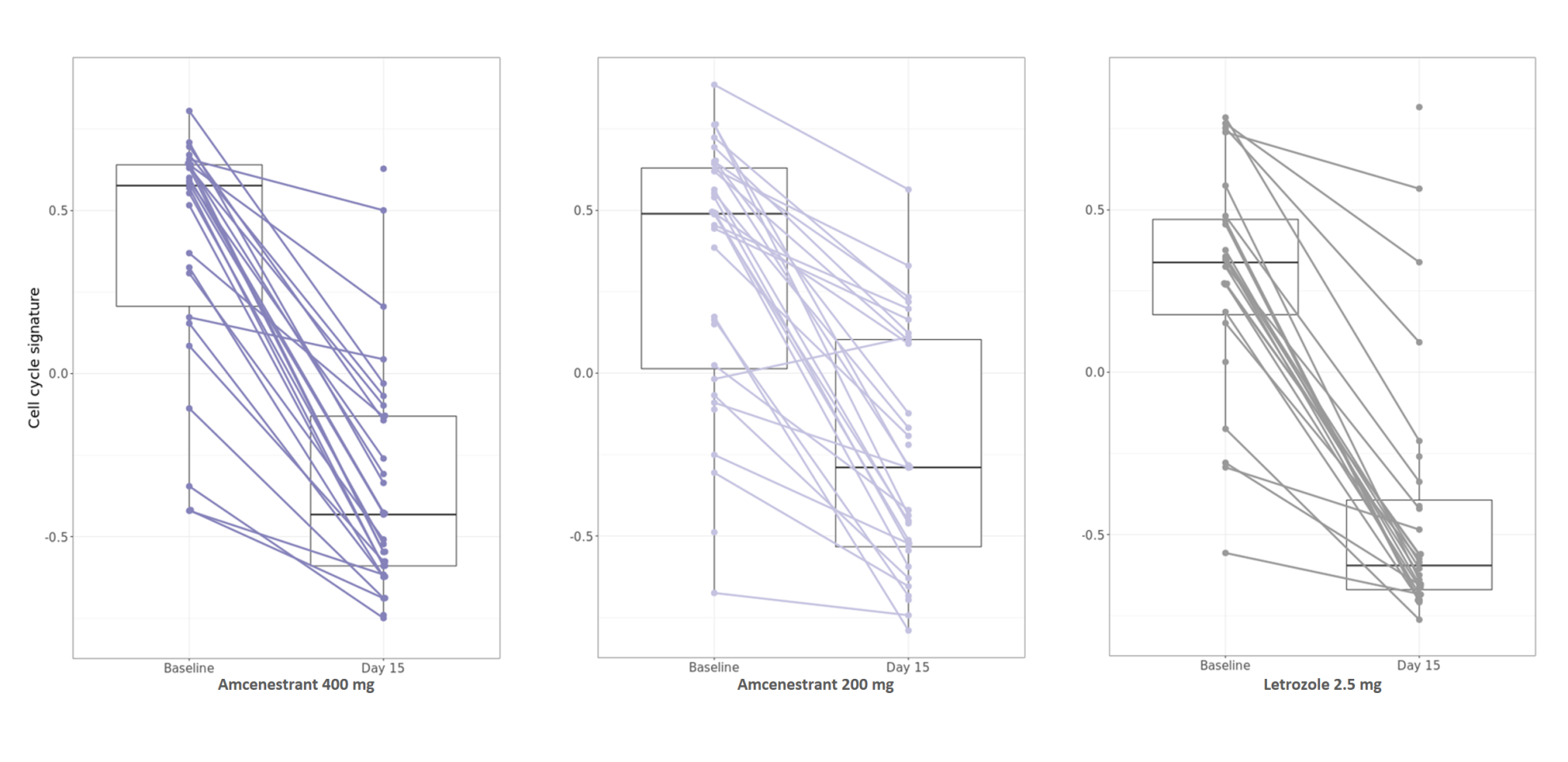


**Figure S4.** **Molecular subtype, assessed using the Prosigna^®^ (PAM50) gene expression assay, at baseline and Day 15.** Abbreviation: HER2, human epidermal growth factor receptor 2.


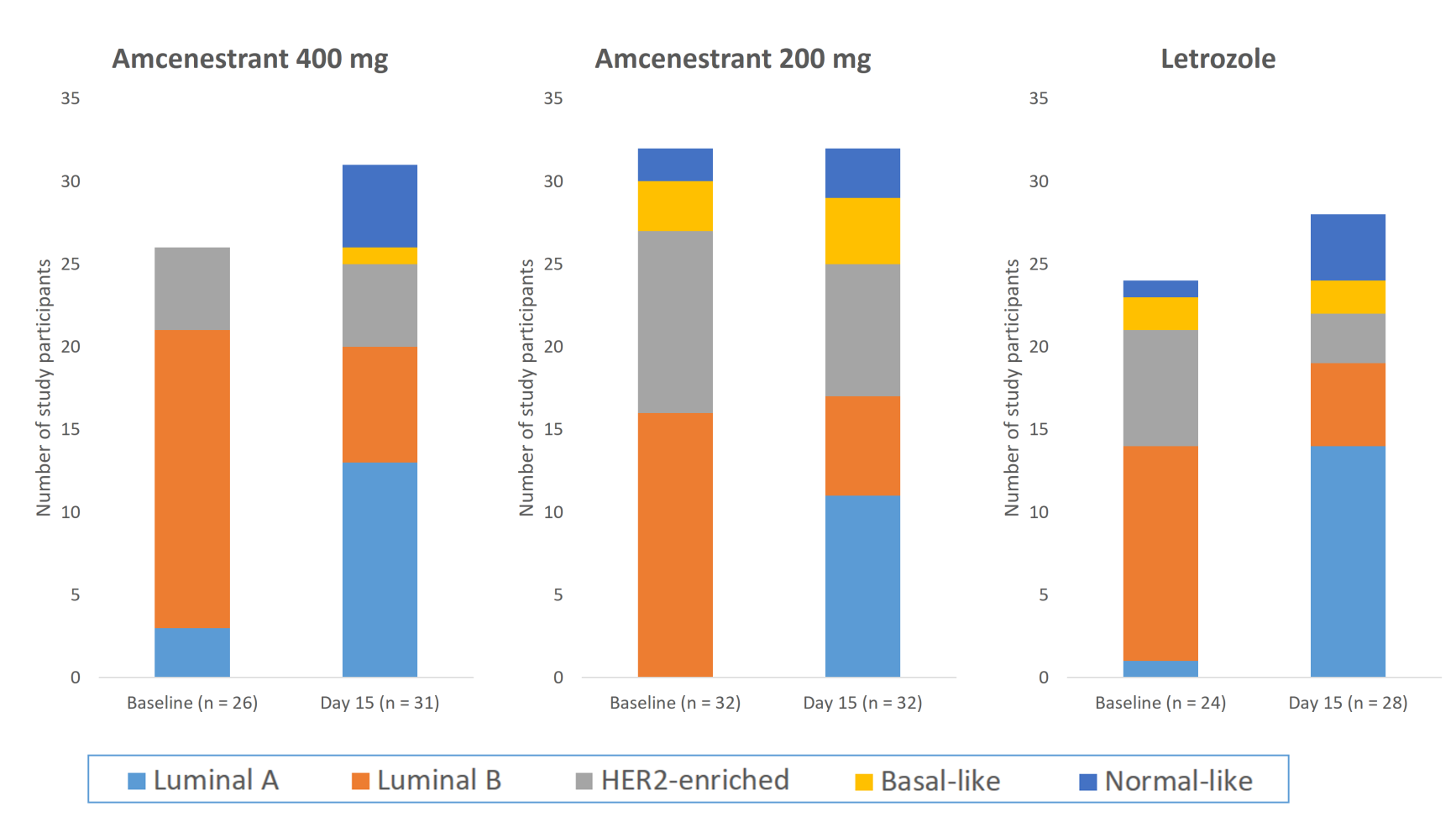


**Figure S5**. **Visualization of the mutational profile at baseline of 75 study participants with available tumor DNA**. SAR439859 = amcenestrant.


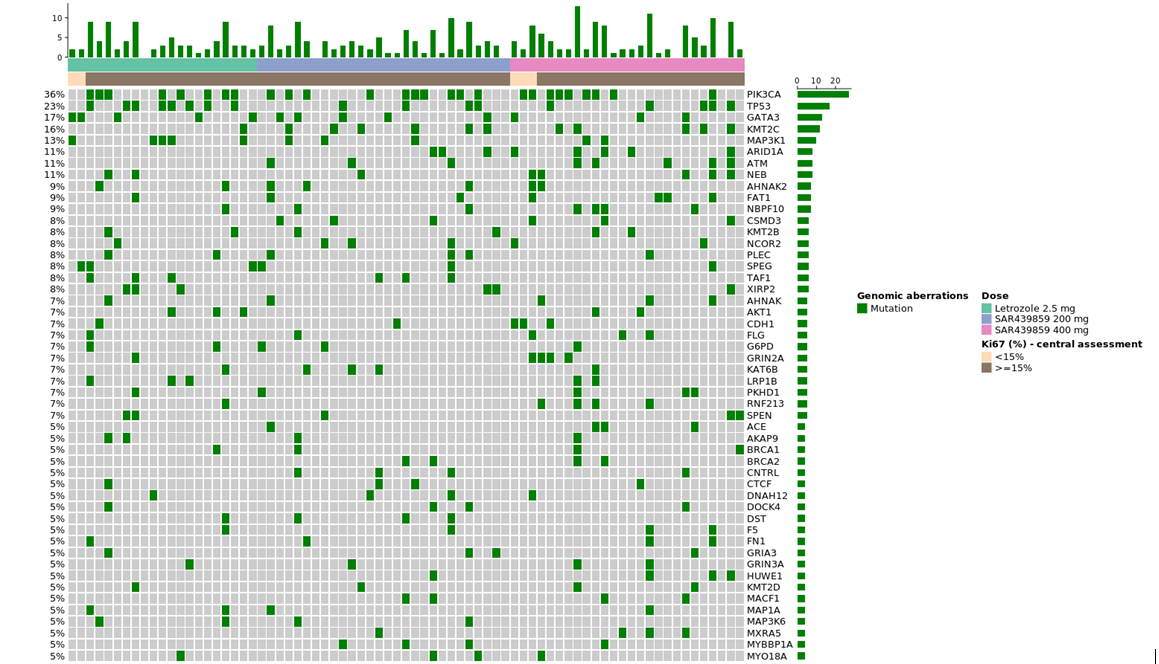


**Figure S6. Visualization of the baseline mutational profile of the 92 study participants with cell-free DNA (cfDNA).** There were 26 participants without mutations and 66 participants with mutations. SAR439859 = amcenestrant.


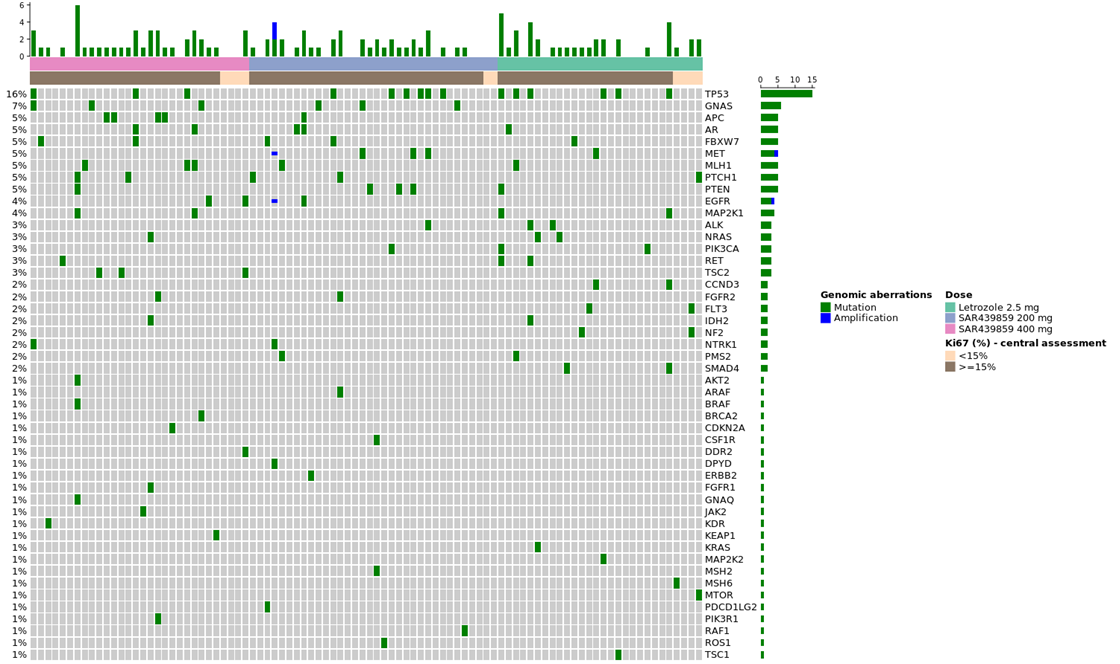

Supplement: Supplementary file 1 — Additional file 1: Campone_AMEERA-4 manuscript_Appendix.docx (supplementary tables and figures) [file 13058_2023_1740_MOESM1_ESM.docx]
